# When the All-Purpose Tool Fails: Manufacturer-Specific Time Dependency of Magnet Mode in Cardiac Implantable Electronic Devices

**DOI:** 10.3390/diagnostics15233056

**Published:** 2025-11-29

**Authors:** Fabienne Kreimer, Dennis Korthals, Julian Wolfes, Christian Ellermann, Florian Doldi, Gerrit Frommeyer, Florian Reinke, Lars Eckardt, Felix K. Wegner

**Affiliations:** Department for Cardiology II: Electrophysiology, University Hospital Munster, 48149 Munster, Germany

**Keywords:** magnet, pacemaker, defibrillator, ICD

## Abstract

**Background:** Previously unreported, the induction of the magnet mode is time-dependent, according to the cardiac implantable electronic device (CIED) manufacturer, directly after device interrogation. The aim of this study was to systematically investigate the response of CIED from all major manufacturers to the application of a magnet. **Methods:** CIED from all manufacturers were utilized and connected to an interactive heart simulator (InterSim III, IB Lang). After the end of CIED interrogation, a CIED magnet was placed over the device, and the response was analyzed. **Results:** Fifteen ICD and eight pacemakers were included. ICDs from the manufacturers Abbott, Boston Scientific, Medtronic and Microport reacted immediately to magnet application by inhibiting antitachycardia function directly after interrogation. In the Biotronik ICD, the magnet mode was only inducible five to seven minutes after the end of the interrogation. In addition, after eight hours of magnet application, the antitachycardia function was automatically and permanently reactivated in all Biotronik ICDs. Pacemakers of Biotronik, Abbott, Boston Scientific, and Microport responded immediately after device interrogation regarding the magnet application. In contrast, Medtronic pacemakers responded only 1.5 min after device interrogation. **Conclusions:** Magnet mode induction directly after CIED interrogation is manufacturer-specific. Our findings might be of importance when performing invasive procedures with devices that cause electrical interference and in palliative care.

## 1. Introduction

Implantable cardiac electronic devices (CIEDs), including implantable cardioverter defibrillators (ICDs) and pacemakers (PMs), are essential in the treatment of patients with cardiac arrhythmias and the prevention of sudden cardiac arrest in patients at increased risk. The number of patients with a CIED will continue to increase over the next years. A key feature of these devices is the ability to interact with magnets that can temporarily alter device functions [1,2]. The CIED magnet mode allows any clinician to interact with the device in a controlled and reversible manner by temporarily inhibiting shock delivery in ICD and inducing specific pacing modes in pacemakers. Understanding the magnet-induced response of various CIEDs is critical for clinicians, especially in situations where magnetic fields could be inadvertently or intentionally applied during medical procedures or when interacting with external devices.

Magnet application is a cornerstone of periinterventional and perioperative management of CIED patients [3,4]. Regarding electromagnetic interference above the umbilicus in patients with PMs, the recent AHA/ACC guideline recommends that either a magnet should be used, or asynchronous mode should be programmed in pacemaker-dependent patients. For ICDs, pacemaker-dependent patients should be programmed to asynchronous mode and the antitachycardia function should be deactivated. For patients who are not pacemaker dependent, the antitachycardia function should be disabled with a magnet [3].

In addition to their therapeutic role, modern CIEDs are important diagnostic tools in cardiology. Continuous intracardiac ECG recording, arrhythmia detection algorithms, and remote monitoring capabilities allow clinicians to diagnose arrhythmias, evaluate therapy effectiveness, and detect device or lead malfunctions [1,2]. The diagnostic accuracy of these systems can, however, be affected by external factors such as electromagnetic interference or magnet application. Understanding how magnet mode interacts with diagnostic data acquisition and storage is therefore essential for clinicians, as inappropriate magnet application may temporarily suspend diagnostic sensing, alter telemetry behavior, or suppress event recording. Hence, clarifying manufacturer-specific differences in magnet response has direct implications not only for therapy management but also for diagnostic reliability.

In previous studies, we observed that magnet mode induction can be time-dependent directly after device interrogation depending on CIED manufacturer [5,6]. To the best of our knowledge, this phenomenon has not been previously reported.

Although the mechanisms underlying these post-interrogation behaviors are not fully understood, several hypothetical explanations may be considered. Variations in post-interrogation telemetry protocols, temporary safety or “handshake” states, and differences in magnet detection circuitry between manufacturers could contribute to the observed latency in magnet-mode activation. Prior studies have shown that manufacturers implement distinct sensing architectures—hall–sensor or reed–switch configurations—and firmware-based filtering algorithms to prevent inappropriate mode switching or electromagnetic interference [1,2,4]. These design differences may plausibly influence how quickly a device resumes full magnet responsiveness after interrogation. While these mechanisms remain speculative, outlining them provides a conceptual framework that may help clinicians understand why the phenomenon might manifest differently across device platforms.

The aim of this study was to systematically investigate the response of CIEDs from all major manufacturers to the application of a magnet directly after device interrogation.

## 2. Methods

**Figure 1:**
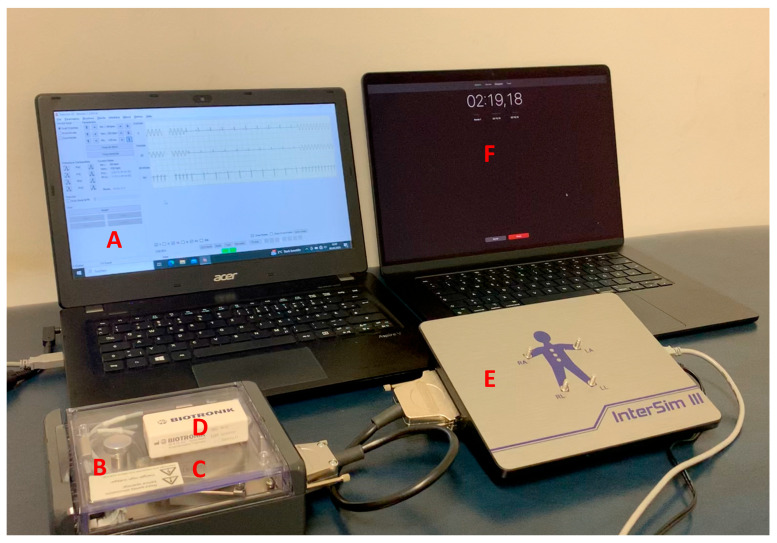
Overview of the experimental setup. (**A**): PC with the InterSim software for programming the arrhythmias; (**B**): adapter box in which the respective CIED (**C**) is connected via the corresponding electrodes. The magnet (**D**) is placed on the adapter box. (**E**): Interactive heart simulator (InterSim III); (**F**): PC with timer. CIED, cardiac implantable electronic device.

In the present study, CIEDs from all major manufacturers were examined (Abbott, Chicago, IL, USA; Biotronik, Berlin, Germany; Boston Scientific, Marlborough, MA, USA; Medtronic, Minneapolis, MN, USA; Microport, Shanghai, China). Ethics approval was not required by the local institutional review board. Devices were programmed according to guideline recommendations [7]. CIEDs were connected to an interactive heart simulator (InterSim III, IB Lang, Rossau, Germany). After interrogation of the ICD and activation of tachytherapies, a CIED magnet of the respective manufacturer was placed over the device after the end of device interrogation. Subsequently, ventricular fibrillation was simulated, and the ICD response was repeatedly analyzed in both the short and long term (Figure 1). Additionally, PM were examined by applying magnets and analyzing the response in the interactive heart simulator to determine if the manufacturer-specific magnet mode of the pacemaker was present [1]. Magnet mode induction was assessed in each CIED, characterized, by audible signals, asynchronous pacing, inability to terminate ventricular tachyarrhythmias, or episodes of magnetic interference recorded in device memory [2].

To ensure comparability across devices, we verified that the manufacturer-supplied magnets were the standard clinical magnets typically used for each system; all magnets were inspected for intact housing and polarity before use and applied at identical distances and orientations. The interactive heart simulator and adapter box do not generate magnetic fields and therefore do not alter magnet detection thresholds; however, we confirmed during preliminary testing that no magnet-related artifacts were induced by the simulator. In addition, all devices operated on the most recent clinically available firmware at the time of testing.

## 3. Results

The present study analyzed 23 CIEDs from all major manufacturers with 15 ICDs (7 Biotronik, 2 Abbott, 2 Medtronic, 2 Boston Scientific, 2 Microport) and 8 PMs (2 Biotronik, 2 Abbott, 2 Medtronic, 1 Boston Scientific, 1 Microport). The analyzed CIEDs are listed in Table 1 and Table 2. All CIEDs were interrogated before and after testing for magnetic interaction, with no change in the relevant measured values being detected for any device.

### 3.1. Magnet Mode in ICD

The ICDs from Abbott, Boston Scientific, Medtronic and Microport reacted immediately to the application of a magnet after the end of the device interrogation by inhibiting the antitachycardia function and shock delivery in ventricular tachyarrhythmias programmed in the interactive heart simulator. None of the seven Biotronik ICDs responded to the magnet application immediately after the device interrogation was completed. In four Biotronik ICD models (Rivacor 5 VR-T DX, Rivacor 3 VR-T, Inlexa 3 VR-T, Itrevia 5 HF-T QP), magnet mode could only be induced five minutes after the end of the interrogation (Supplemental Videos S1 and S2). With three other Biotronik ICD models (Iforia 3 HF-T, Iforia 3 DR-T, Iforia 5 VR-T DX), magnet mode could only be initiated seven minutes after the end of the interrogation. During this period, the antitachycardia stimulation and shock delivery continued despite correct application of a manufacturer-specific CIED magnet. Results were consistent even if the magnet was removed and re-applied during this timeframe, indicating that respective devices were completely unresponsive to magnet application.

**Figure 2:**
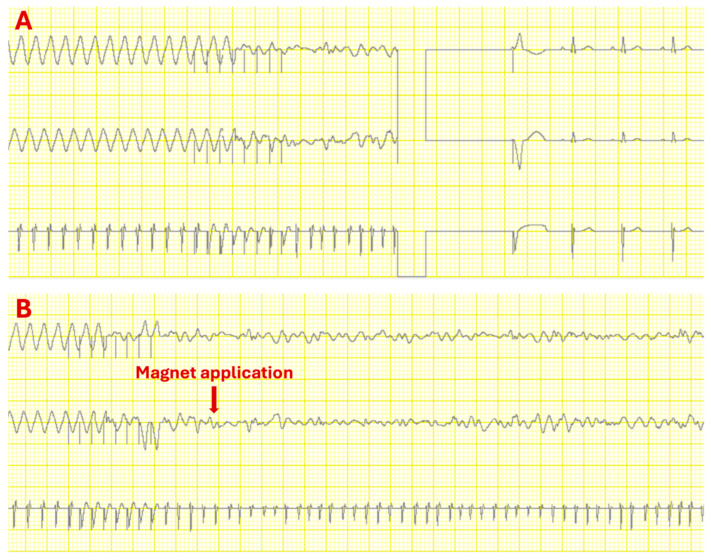
Excerpts from the Interactive Heart Simulator. An ICD from Abbott was used. (**A**): No magnet application. The ICD responds with antitachycardia pacing to the ventricular tachycardia, which then degenerates into ventricular fibrillation, triggering an ICD shock. Afterward, a normal sinus rhythm is observed. (**B**): With magnet application. The ICD responds with antitachycardia pacing to the ventricular tachycardia, which then degenerates into ventricular fibrillation. However, a magnet is now applied, and no further antitachycardia function occurs.

In addition, there was a difference between the manufacturers after eight hours of magnet application: In all Biotronik ICDs, the antitachycardia function was automatically and permanently reactivated. In contrast, the ICDs from Abbott, Medtronic, Boston Scientific and Microport remained inhibited even after eight hours of magnetic application, and no shocks were delivered (Figure 2, Table 1).

### 3.2. Magnet Mode in Pacemakers

**Figure 3:**
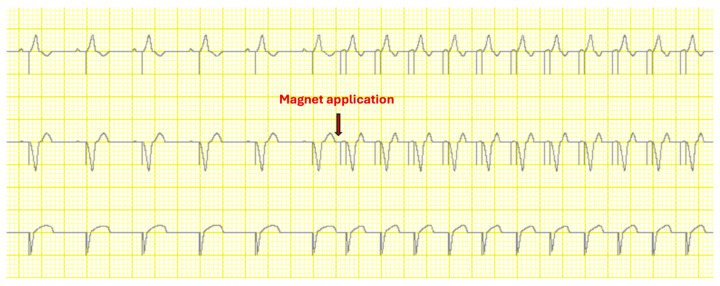
Excerpt from the Interactive Heart Simulator. A Boston Scientific pacemaker was used. After magnet application, a fixed-rate pacing occurs with a frequency of around 100 beats per minute, which corresponds to the typical magnet response in pacemakers from Boston Scientific.

The PM from Biotronik, Abbott, Boston Scientific and Microport responded to the application of a magnet immediately after the device interrogation. In contrast, the Medtronic PM did not respond immediately; instead, a delay of 1.5 min after the end of the device interrogation was required for the magnet to initiate a response (Figure 3, Table 2).

## 4. Discussion

In this study, temporal differences in magnet mode induction of CIEDs from all major manufacturers shortly after device interrogation were systematically investigated for the first time. The main results were:

(1) The ICDs from Abbott, Boston Scientific, Medtronic, and Microport immediately inhibited their antitachycardia function after the application of the magnet, while the ICDs from Biotronik showed a delayed response, with the magnet mode being triggered 5 to 7 min after the end of device interrogation.
(2) After eight hours of magnet application, all Biotronik ICDs automatically reactivated the antitachycardia function, while ICDs from other manufacturers remained inactive.
(3) Biotronik, Abbott, Boston Scientific, and Microport pacemakers responded to a magnet immediately after interrogation, while the Medtronic pacemakers showed a delay of 1.5 min.

Interestingly, to the best of our knowledge, the inhibition of magnet mode induction for a fixed time after CIED interrogation has not been reported by either respective manufacturers or independent researchers. By systematically analyzing devices from all major manufacturers, we have demonstrated significant differences in the temporal dynamics of magnet mode induction that may have clinical implications for patient management and procedure planning [4,8,9]. Beyond the therapeutic implications, our findings have diagnostic relevance. Since CIEDs serve as both therapeutic and diagnostic devices, delayed or absent magnet responses can impact diagnostic reliability during perioperative monitoring. For instance, during procedures involving electrocautery, magnet use is not only meant to inhibit therapy but also to ensure diagnostic accuracy by preventing inappropriate detection of electromagnetic interference as arrhythmic events. A delayed magnet response may cause inappropriate event labeling or compromise the accuracy of diagnostic recordings. Our results highlight the variability in manufacturer-specific responses to magnet application, which may be relevant in situations where device interactions occur during medical procedures or other interventions.

### 4.1. Delayed Response of Biotronik ICDs

One of the main findings of this study was the delayed response of Biotronik ICDs to the application of a magnet shortly after the end of device interrogation. In contrast to devices from other manufacturers (Abbott, Boston Scientific, Medtronic and Microport), which inhibited shock delivery and antitachycardia pacing during magnet application immediately after device interrogation, Biotronik ICDs did not respond until five to seven minutes after device interrogation. Notably, this delayed response was apparent in all tested Biotronik ICD models, including the Rivacor, Inlexa, Itrevia and Iforia series. During this delay, these devices continued to deliver antitachycardia pacing and shock therapy despite the application of the magnet. This delayed response could be due to differences in the underlying hardware or software programming between manufacturers, which could be designed to prevent unintentional discontinuation of therapy or premature deactivation in case of accidental magnet exposure [2,10]. However, an immediate inhibition of therapy, which is necessary in surgical interventions, is not possible after interrogation [11,12].

### 4.2. Reactivation of the Antitachycardia Function in Biotronik ICDs

Another important finding of this study was the automatic reactivation of the antitachycardia function in all analyzed Biotronik ICDs after 8 h of continuous magnetic application. In contrast, ICDs from other manufacturers (Abbott, Medtronic, Boston Scientific and Microport) remained inhibited, with shock delivery still suppressed after 8 h of magnetic application. The reactivation of therapy in Biotronik ICDs could have implications for palliative care, where maintaining magnet mode may help to avoid unnecessary shocks or stimulation. In addition, this automatic reactivation must be considered during extended surgical procedures [13,14,15,16]. In both scenarios, the magnets must be removed after 8 h of exposure and re-applied to ensure that the ICD remains inhibited.

### 4.3. Responses for Pacemakers

The response of PMs to the magnet application was more consistent across manufacturers. PMs from Biotronik, Abbott, Boston Scientific and Microport all responded immediately after device interrogation. In contrast to the Medtronic PMs, which responded to the magnet with a delay of 1.5 min. The delayed response in the Medtronic PMs is less pronounced than the delayed response in the Biotronik ICDs but nevertheless demonstrates that there is a time-dependent response between the different manufacturers.

### 4.4. Clinical Relevance of the Findings

While the overall relevance of the observed differences in magnet mode responses between manufacturers in routine clinical practice may be limited, the observed temporal differences in the response of Biotronic ICDs to magnet application are important in certain clinical scenarios such as perioperative management. An important aspect is the delay in magnet response of Biotronic ICDs, which do not respond to the magnet until 5–7 min after device interrogation. Therefore, this is essential for surgical procedures or interventions that may require the use of electrical devices such as electrocautery. Knowledge of manufacturer-specific delays is essential for planning the timing of magnet use, particularly when electrocautery or other sources of electromagnetic interference are expected. For Biotronik ICDs, the 5–7 min delay after interrogation means that immediate therapy inhibition cannot be assumed, requiring clinicians to either avoid recent interrogations before surgery or to allow sufficient time for magnet mode to become active before initiating procedures.

Another important aspect related to the magnetic response of the Biotronik ICDs described in our study is the reactivation of antitachycardia functions after 8 h of magnet application.

Deactivation of tachytherapies is common in palliative care and may be achieved by magnet application when device interrogation is not immediately obtainable [17]. In these situations, magnet use offers a rapid means of preventing potentially distressing shocks in patients approaching the end of life, particularly when urgent reprogramming by a device specialist is not feasible. When doing so in Biotronik ICD, being aware that the magnet must be removed and reapplied within 8 h is critical to prevent the device from reactivating the antitachycardia function and possibly causing unnecessary discomfort at the end of a patient’s life. This time-limited suppression of therapy is unique to Biotronik ICDs among the tested manufacturers and introduces an additional layer of complexity for palliative care teams, who may reasonably expect continuous inhibition of tachytherapies during prolonged care episodes. In practical terms, caregivers would need to ensure that the magnet remains securely in place, continuously monitor the cumulative duration of magnet application, and reapply the magnet after 8 h to maintain therapy inhibition. However, repeated removal and reapplication of the magnet solely for tracking the elapsed time is impractical in real-world palliative settings. For this reason, deactivation of tachytherapies within an 8 h window is likely the most reliable and patient-centered approach for Biotronik devices. This strategy minimizes the risk of unintentional shock delivery or antitachycardia pacing—which can cause considerable distress, especially in patients with advanced illness and limited physiological reserve. Consequently, clear communication and coordinated planning between cardiology teams, palliative care providers, and family members are essential to ensure uninterrupted patient comfort and to prevent unexpected reactivation of device therapies during end-of-life care.

Furthermore, magnet-induced responses can alter the diagnostic interpretation of stored electrograms and event markers. If magnet application fails to suspend therapies and electromagnetic noise is recorded as ventricular fibrillation or tachyarrhythmias, diagnostic data may be misrepresentative.

## 5. Limitations

This study was conducted in a controlled laboratory setting using an interactive heart simulator, which may not entirely reflect the complex clinical real-world setting. Moreover, we decided to conduct experiments with a representative selection of CIEDs from all major device manufacturers, as we propose that the results are transferable to other comparable CIEDs. Nevertheless, we cannot rule out the possibility that individual CIEDs that were not tested may react differently to the magnet application.

## 6. Conclusions

This study is the first to systematically investigate the manufacturer-specific time dependence of magnetic mode induction in CIEDs. Our results reveal that Biotronik ICDs exhibit a delayed response to magnet application directly after device interrogation, in contrast to ICDs from other manufacturers, which respond immediately. In addition, Medtronic pacemakers had a delayed response time of 1.5 min to magnet application after device interrogation, whereas pacemakers from other manufacturers react immediately. These results emphasize the importance of understanding the different response times of different device manufacturers, especially in clinical settings with invasive procedures or in situations with potential electrical interference. Clinicians should be aware that Biotronik ICDs may not respond to a magnet until five to seven minutes after the end of device interrogation. In addition, reactivation of tachytherapies in Biotronik ICDs after eight hours of magnet application can have significant implications, particularly in palliative care settings.

## Figures and Tables

**Table 1:** **Overview of the included ICD.** Green: inhibition of antitachycardia function despite magnet application; yellow: no inhibition of antitachycardia function despite magnet application.

		Time After the End of Device Query Long Time															
	CIED Name & Type	0.5 min	1 min	1.5 min	2 min	2.5 min	3 min	3.5 min	4 min	4.5 min	5 min	5.5 min	6 min	6.5 min	7 min	7 h	8 h
Med-tronic	Mirro VR (VVI-ICD)																
	Compia Quad (CRT-D)																
Abbott	Ellipse VR (VVI-ICD)																
	Unify Assura (CRT-D)																
Biotronik	Rivacor 5 VR (VVI-ICD)																
	Rivacor 3 VR (VVI-ICD)																
	Iforia 3 HF (CRT-D)																
	Iforia 3 DR (DDD-ICD)																
	Iforia 5 VR (VVI-ICD)																
	Inlexa 3 VR (VVI-ICD)																
	Itrevia 5 HF (CRT-D)																
Micro-port	Paradym VR (VVI-ICD)																
	Paradym 2 DR (DDD-ICD)																
Boston Sci.	Origen Mini (VVI-ICD)																
	Teligen 100 (DDD-ICD)																

**Table 2:** **Overview of the included pacemakers.** Green: magnet mode induction; yellow: no magnet mode induction possible. CIED, cardiac implantable electronic device.

		Time After the End of Device Query									
	CIED Name & Type	0.5 min	1 min	1.5 min	2 min	2.5 min	3 min	3.5 min	4 min	4.5 min	5 min
Medtronic	Sensia DR (DDD-PM)										
	Relia SR (single chamber)										
Abbott	Endurity Core (DDD-PM)										
	Endurity (single chamber)										
Biotronik	Ecuro DR (DDD-PM)										
	Amvia Edge DR (DDD-PM)										
Micro-port	Reply DR (DDD-PM)										
Boston Sci.	Visionist (CRT-P)										

## Data Availability

Data are contained within the article or Supplementary Material.

## Supplementary Materials

The following supporting information can be downloaded at: https://www.mdpi.com/article/10.3390/diagnostics15233056/s1, Video S1: Experimental setup as described in Figure 1. A ventricular tachycardia is simulated approximately 2.5 min after the end of the device interrogation and subsequent magnet application. A shock is delivered regardless of the magnet application. Video S2: Experimental setup as described in Figure 1. A ventricular tachycardia is simulated approximately 5.5 min after the end of the device interrogation and subsequent magnet application. At this point, the magnet application no longer results in shock delivery.
